# Potential of a constitutive-UPR and histone deacetylase A-deficient *Saccharomyces cerevisiae* strain for biomolecule production

**DOI:** 10.1128/aem.00644-25

**Published:** 2025-08-07

**Authors:** Yuki Ishiwata-Kimata, Phuong Thi Mai Nguyen, Maya Sugimoto, Yukio Kimata

**Affiliations:** 1 Division of Biological Science, Graduate School of Science and Technology, Nara Institute of Science and Technology 98337 , Ikoma, Nara, Japan; Chalmers tekniska hogskola AB, Gothenburg, Sweden

**Keywords:** unfolded protein response, protein secretion, endoplasmic reticulum, yeast

## Abstract

The endoplasmic reticulum (ER) is an organelle responsible for the production of secretory proteins and lipid molecules. In many ascomycetous yeasts, including *Saccharomyces cerevisiae*, *HAC1* mRNA undergoes splicing to yield *HAC1*i mRNA (“i” denotes induced), which is translated into the nuclear transcription factor Hac1 upon ER dysfunction. The extensive transcriptome change triggered by Hac1 is called the unfolded protein response (UPR), which leads to ER enforcement. As an unregulated UPR continuously elevates ER activity and size, *S. cerevisiae* cells exhibit increased production of secretory proteins and lipid molecules when Hac1 is constitutively and artificially expressed. While this technique holds promise for industrial bioproduction, it has a notable drawback, as cells strongly expressing Hac1 exhibit slow growth, making it difficult to handle and store them. To address this issue, in this study, we searched for fast-growing mutants of *HAC1*i cells carrying the *HAC1*i sequence at the *HAC1* locus to express Hac1 constitutively. We found that histone deacetylase A (HDA)-deficient mutations, including *Δhda3*, accelerated the growth of Hac1-expressing cells. Despite their increased growth rate, *Δhda3HAC1*i cells showed stronger expression of prominent UPR target genes related to ER function than *HAC1*i cells. Moreover, *Δhda3HAC1*i cells carried a structurally complex and expanded ER and exhibited high-yield production of triglycerides and of different heterologous model biomolecules, β-carotene and Taka amylase A. In conclusion, we propose that combining artificial Hac1 expression with mutations that affect the chromosomal status holds promise for optimizing yeast-based bioproduction systems.

**IMPORTANCE:**

The production of commercially valuable biomolecules using genetically modified *Saccharomyces cerevisiae* is an important biotechnological process that has been partly industrialized. Because secretory proteins and many lipid molecules are produced in and/or from the endoplasmic reticulum (ER), their production is expected to be improved by artificial enhancement of ER functions. This can be accomplished by artificial and constitutive expression of the nuclear transcription factor Hac1. In wild-type cells, Hac1 is induced upon ER dysfunction and upregulates its functions. A major drawback of cells artificially overexpressing Hac1 is their slow-growing phenotype. Here, we showed that the growth of artificially Hac1-expressing cells is fastened by histone deacetylase A-deficient mutations, leading to efficient biomolecule production. Our study provides an intriguing example of how the properties of a genetically modified yeast strain can be improved by a mutation that alters its chromosomal status.

## INTRODUCTION

Eukaryotic cells commonly carry flat or tubular-shaped intracellular sacs, namely the endoplasmic reticulum (ER). While the ER expands throughout the cytosol in a wide variety of animal and plant cells, yeast cells seem to carry less spread ER, which is composed of the cortical ER and nuclear envelope (1, 2). The ER acts as a site for folding and modification of secretory proteins, including N-linked glycosylation. Moreover, various lipid molecules are biosynthesized on the ER membrane. Dysfunction or functional shortage of the ER is referred to as ER stress and is frequently accompanied by the accumulation of unfolded proteins in the ER.

The unfolded protein response (UPR) is a cytoprotective transcriptome shift induced by ER stress. While UPR has been observed in various eukaryotic species, the intracellular signaling pathway was initially discovered through frontier studies using *Saccharomyces cerevisiae* as a model organism (3). Ire1 is an ER-localized transmembrane protein that serves as an ER stress sensor. Upon ER stress, Ire1 self-associates and exerts a potent endoribonuclease activity. In many ascomycetous fungal species, including *S. cerevisiae*, *HAC1* mRNA is a substrate of Ire1. The *HAC1* gene is transcribed as an intron-containing form, namely *HAC1*u mRNA (“u” stands for uninduced). Under ER stress conditions, Ire1 promotes splicing of *HAC*1u mRNA to yield the intronless form, namely *HAC1*i mRNA (“i” stands for induced), which is translated into the active transcription-factor protein Hac1 (4).

Genome-wide transcriptome analyses by us and others have revealed that hundreds of genes are regulated by Hac1 in *S. cerevisiae* (5, 6). The genes upregulated by the Ire1- and *HAC1*-dependent UPR, namely, the UPR target genes, include those encoding proteins related to ER protein folding, modification, and flux. This observation explains the role of the UPR in elevating ER function during ER stress. According to Schuck et al. (7), the UPR causes ER expansion by inducing genes related to lipid biosynthesis.

In addition to another yeast species, *Komagataella phaffii* (syn*. Pichia pastoris*), *S. cerevisiae* often serves as a platform for producing heterologous secretory proteins used as biopharmaceuticals and industrial enzymes (8). Moreover, many efforts have been devoted to genetically engineering *S. cerevisiae* to produce commercially valuable lipid molecules (9, 10). Since the ER is the site where these biomolecules are biosynthesized, it is anticipated that their productivity can be increased by enhancing ER function through artificial induction of the UPR.

Cells expressing the intronless mutant *HAC1* gene (*HAC1*i), from which Hac1 is translated, exhibit a non-regulated UPR. Hereafter, this genetic manipulation is referred to as artificial Hac1 expression. As initially demonstrated by Valkonen et al. (11), artificial Hac1 expression from the promoter of a housekeeping gene results in a high production of secretory proteins in *S. cerevisiae* cells. Similar strategies have been used to improve the quantity and quality of other heterologous secretory proteins produced by *S. cerevisiae* and other yeast species (12–17). Kim et al. (13) reported that the biological activity of insulin produced by a Hac1-expressing *S. cerevisiae* strain was higher than that of insulin produced by the control strain. Moreover, *S. cerevisiae* cells artificially expressing Hac1 exhibit high production of lipid molecules (18–20).

It is likely that the ability of artificial Hac1 expression to increase biomolecule production depends on its expression level (11, 20). To achieve a high production of secretory proteins or lipid molecules, *S. cerevisiae* cells must strongly express Hac1, leading to potent UPR induction. Nevertheless, it should also be noted that the constitutive and strong Hac1 expression severely impairs cell growth (4, 19–21). This results in a reduction in total product yield. Furthermore, when artificially Hac1-expressing cells are continuously grown, rapidly growing progenies that have lost the ability to induce the UPR get dominant in cultures (19).

In this paper, we propose a strategy to overcome this problem. Growth retardation of artificially Hac1-expressing cells was partly but substantially rescued by knockout mutations of genes encoding histone deacetylase A (HDA) subunits. We demonstrated the potential of the genetic engineering strategy developed here for high-yield production of lipids and secretory proteins.

## RESULTS

### HDA deficiency accelerates the growth of artificially Hac1-expressing cells without impairing their UPR-inducing ability

As in our previous studies (6, 19), we introduced the intronless *HAC1*i mutation into the *HAC1* gene of the *S. cerevisiae ire1*-knockout (*Δire1*) strain Y11907 using the pop-in/pop-out mutation technique (refer to the Materials and Methods for details). Some progenies that carried the original *HAC1* gene, namely *HAC1*u cells (*Δire1*/wild-type *HAC1*), were unable to convert *HAC1*u mRNA to *HAC1*i mRNA (6). Other progenies that successfully deleted the *HAC1* intron sequence in their genome, namely *HAC1*i cells (*Δire1*/*HAC1*i), constitutively expressed *HAC1*i mRNA and exhibited an unregulated UPR (6).

**Fig 1:**
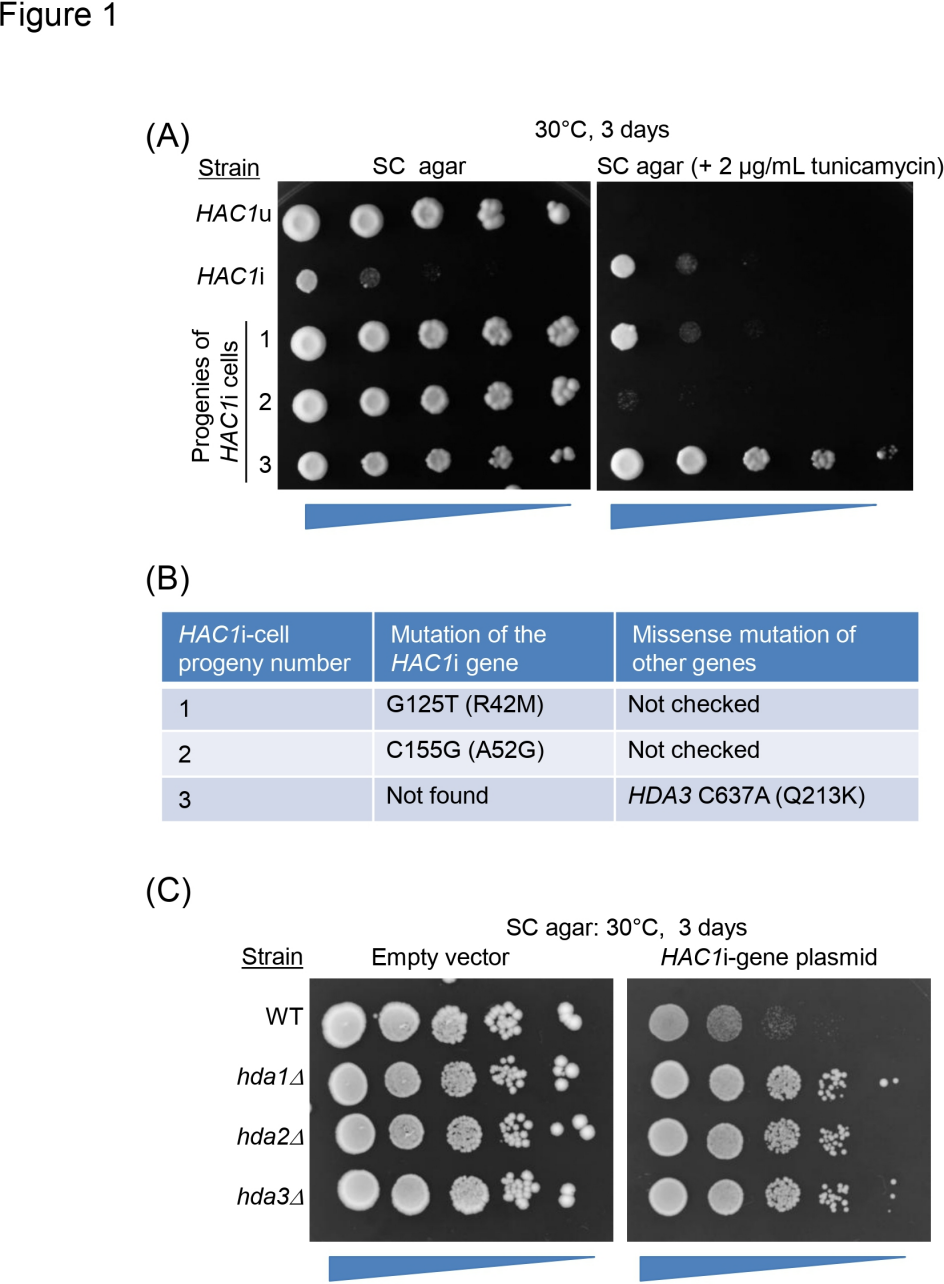
Isolation of fast-growing progenies of *HAC1*i cells. (**A**) *HAC1*u cells, *HAC1*i cells, and three fast-growing spontaneous mutants of *HAC1*i cells (progenies 1 to 3) were subjected to a spot assay to monitor their growth on the indicated agar plates. Unlike progenies 1 and 2, progeny 3 maintained the tunicamycin resistance phenotype. (**B**) Mutations found in progenies 1–3 are indicated. (**C**) Wild-type (WT; BY4741) strain and its kanMX-based gene-deletion mutants were transformed with the *HAC1*i-gene plasmid (pRS316Hac1-238) or control empty vector (pRS316), and subjected to a spot assay to monitor their growth on SC agar plates. Unlike wild-type cells, HDA-deficient (*hda1Δ*, *hda2Δ,* and *hda3Δ*) mutants grew quickly, even when carrying the *HAC1*i-gene plasmid.

Reproducing our previous observations (19), *HAC1*i cells grew much slower than *HAC1*u cells on synthetic complete (SC) agar plates (Fig. 1A). On the other hand, *HAC1*i cells, but not *HAC1*u cells, grew in the presence of tunicamycin, which acts as an N-glycosylation inhibitor and induces potent ER stress, because *HAC1*u cells cannot provoke the UPR (Fig. 1A) (6).

As described in the Introduction, the slow-growing phenotype of *HAC1*i cells is unstable. In the present study, we spread *HAC1*i cells on SC agar plates and picked fast-growing colonies. Three clones that stably exhibited a fast-growing phenotype were deduced to be spontaneous mutants of *HAC1*i cells and named progenies 1, 2, and 3 (Fig. 1A). Unlike progenies 1 and 2, progeny 3 grew well even in the presence of tunicamycin (Fig. 1A). This observation suggests that the UPR was not abolished in progeny 3.

Next, we searched for the mutated genes in the mutant progenies. The *HAC1*i gene was PCR-amplified and subjected to Sanger sequencing. As shown in Fig. 1B, we found missense mutations in the *HAC1*i gene in progenies 1 and 2. We assumed that these were functionless (or almost functionless) mutations of the *HAC1i* gene, which impaired the UPR and caused the fast-growing phenotype. In contrast, the *HAC1*i gene in progeny 3 did not have any mutations. We then subjected the parent *HAC1i* strain and progeny 3 to full-genome sequencing and found a missense mutation in the *HDA3* gene (Fig. 1B).

The product protein of *HDA3* is known to form the HDA complex with *HDA1* and *HDA2* (22–24). Therefore, we presumed that HDA deficiency accelerates the growth of cells harboring the *HAC1*i gene. To confirm this idea, we transformed *S. cerevisiae* cells with a plasmid carrying the *HAC1*i gene with its authentic 5′- and 3′-untranslated regions (UTRs), namely pRS316Hac1-238. Hac1 was expressed from pRS316Hac1-238 under the control of the authentic *HAC1* promoter. As shown in Fig. 1C, the growth of wild-type cells was severely retarded by the introduction of the *HAC1*i-gene plasmid, whereas that of the HDA-deficient mutants was not.

For further investigation, we introduced a markerless knockout mutation of the *HDA3* gene, *hda3Δ0*, into *S. cerevisiae* to obtain *hda3Δ0* cells, *hda3Δ0HAC1*u cells (*hda3Δ0*/*Δire1*/wild-type *HAC1*), and *hda3Δ0HAC1*i cells (*hda3Δ0*/*Δire1*/*HAC1*i). In the experiment shown in Fig. S1, cell lysates were analyzed by western blotting to detect acetylated and total histone H3 levels. Histone H3 is known to be the prominent target of HDA (22). As expected, the *hda3Δ0* mutation appeared to increase the acetylation level of histone H3 in both *HAC1*i cells and *HAC1*u cells.

**Fig 2:**
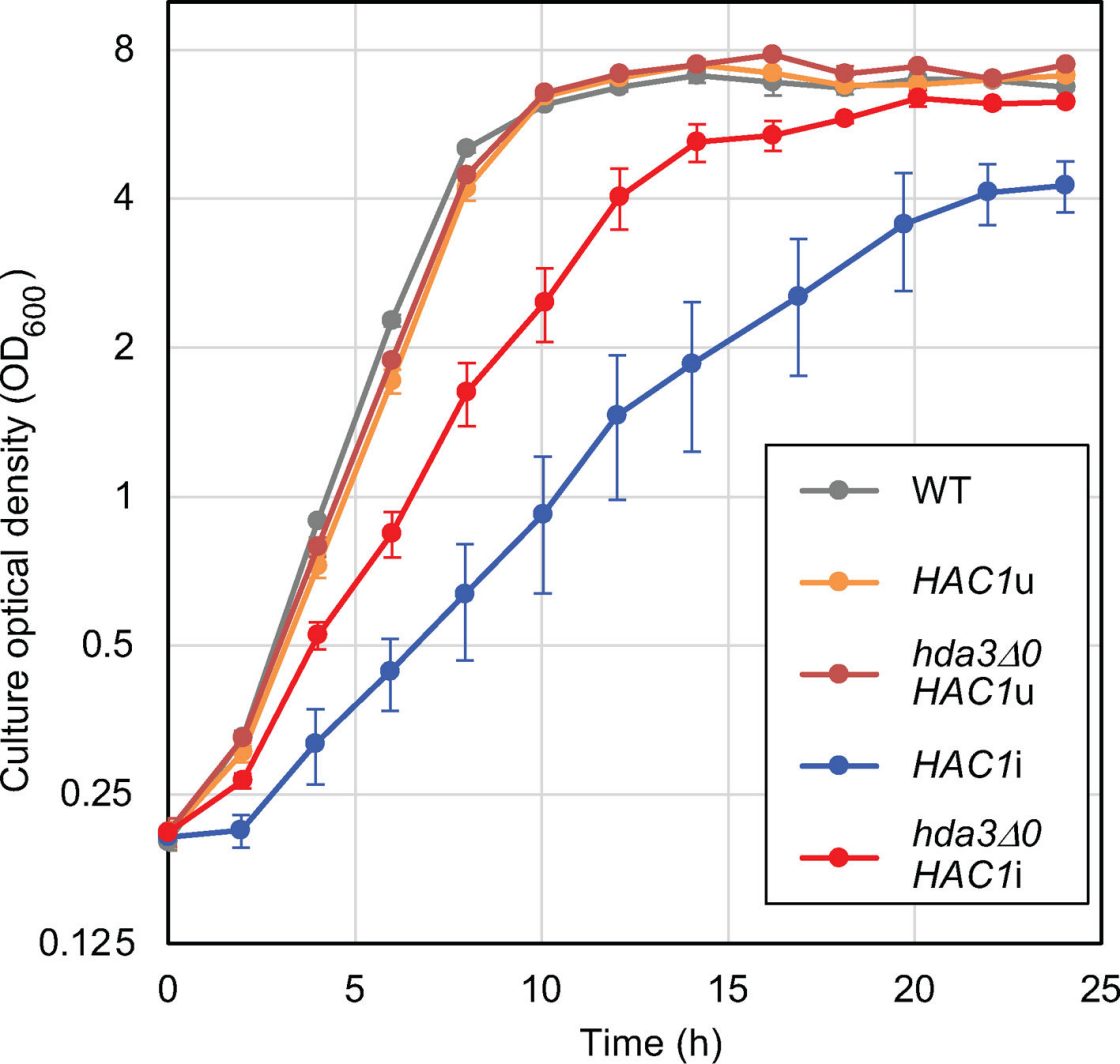
Growth properties of cells carrying or not carrying the *HDA3* deletion mutation. Wild-type (WT; BY4742) cells and their derivatives were grown in SC liquid medium, and the optical density of the cultures was monitored. While wild-type and *HAC1*u cells grew almost equally in both the presence and absence of the *HAC1* gene, *hda3ΔHAC1*i cells grew faster than *HAC1*i cells.

In the experiment shown in Fig. 2, cells were cultured in liquid SC medium to monitor their growth. The *hda3Δ0* mutation did not considerably alter the growth of wild-type or *HAC1*u cells. In contrast, *hda3Δ0HAC1*i cells grew faster than *HAC1*i cells, albeit slower than the other strains. It should also be noted that, unlike *HAC1*i cells, *hda3Δ0HAC1*i cells reached a final culture density comparable to that of the other strains.

HDA is known to control the gene expression in *S. cerevisiae* (25, 26). Thus, we investigated the transcriptome changes caused by the *hda3Δ0* mutation using mRNA-seq analysis of *HAC1*u, *HAC1*i, *hda3Δ0HAC1*u, and *hda3Δ0HAC1*i cells. Table S1 presents the raw data obtained from this analysis.

**Fig 3:**
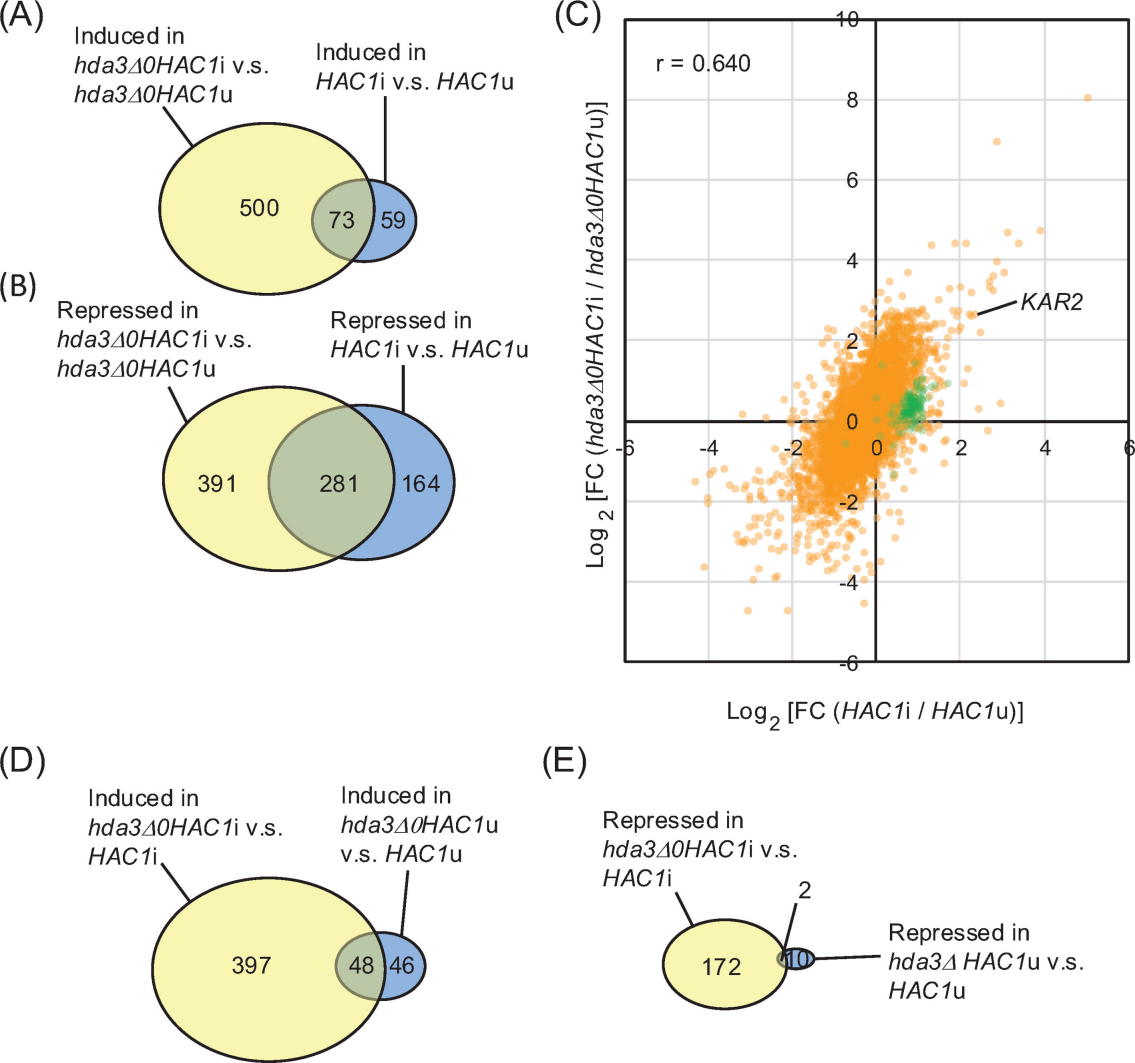
Transcriptome shift induced by the *HAC1*i and *hda3Δ0* mutations. Cells were incubated at 30°C in SC liquid medium, and RNA samples were extracted from growing-phase cultures for mRNA-seq analysis. (**A and B**) Differentially expressed genes (DEGs; fold change (FC) in transcripts per million [TPM] <0.5 or >2.0) between the two cell types (*hda3Δ0HAC1*i vs *hda3Δ0HAC1*u and *HAC1*i vs *HAC1*u) were counted and are presented as Venn diagrams. (**C**) FC in TPM between the indicated cell types is presented as a scatter plot. Both the x- and y-axes represent Log_2_ of the FC. Each dot represents one gene. *KAR2* is a prominent UPR target gene. Ribosomal protein genes (*RPL*, *RPP*, and *RPS*) are indicated by green dots. (**D and E**) Differentially expressed genes (DEGs; fold change (FC) in TPM <0.5 or >2.0) between the two cell types (*hda3Δ0HAC1*i vs *HAC1*i and *hda3Δ0HAC1*u vs *HAC1*u) were counted and are presented as Venn diagrams. In the Venn diagrams shown in A, B, C, and D, genes with high, but not statistically significant, expression changes (*P* > 0.05, FC <0.5 or >2.0) were eliminated from the analyses. The *HAC1*i mutation differentially expressed more genes in the presence than in the absence of the *hda3*Δ mutation. Likewise, the *hda3*Δ mutation differentially expressed more genes in the presence than in the absence of the *HAC1*i mutation. However, many ribosomal protein genes appeared to be upregulated by the *HAC1*i mutation only in the absence of the *hda3*Δ mutation.

Consistent with our previous observation (6), a fair number of genes were differentially expressed (induced or repressed) between *HAC1*u and *HAC1*i cells (Fig. S2A). The comparison between *hda3Δ0HAC1*u and *hda3Δ0HAC1*i cells showed the differential expression of more genes than between *HAC1*u and *HAC1*i cells (compare Fig. S2A and B; Fig. 3A and B). Figure 3C shows that the difference in gene expression between *hda3Δ0HAC1*u and *hda3Δ0HAC1*i cells correlated with the difference between *HAC1*u and *HAC1*i cells (correlation efficiency = 0.640). Therefore, we deduced that, overall, Hac1 normally induces (or represses) UPR target genes, even when cells carry the *hda3Δ0* mutation. This observation supports our idea that the HDA-deficient mutation accelerates the growth of *HAC1*i cells without impairing UPR induction.

Histone deacetylation leads to chromatin condensation, which generally represses gene expression (27). In agreement with this insight, the *hda3Δ0* mutation induced a fair number of genes in *HAC1*u cells (Fig. S2C). The comparison between *HAC1*i and *hda3Δ0HAC1*i cells showed the differential expression of more genes than between *HAC1*u and *hda3Δ0HAC1*u cells (compare Fig. S2C and D; Fig. 3D and E).

Based on the observations presented thus far, we presume that the *HAC1*i mutation affects the gene expression profile more strongly when cells carry an HDA-deficient mutation. Nevertheless, it should also be noted that, although not large in number, there are genes differentially expressed between *HAC1*u and *HAC1*i cells, but not between *hda3Δ0HAC1*u and *hda3Δ0HAC1*i cells (blue areas in Fig. 3A and B). Notably, among the 59 genes in the blue area in Fig. 3A, 26 genes were ribosomal protein genes (Table S2: *RPL* genes, *RPS* genes, *RPP1B*, and *RPP2A*). Figure 3C also indicates that a large portion of the ribosomal protein genes (green-colored dots) were induced more by the *HAC1*i mutation in the absence than in the presence of the *hda3Δ0* mutation.

**Fig 4:**
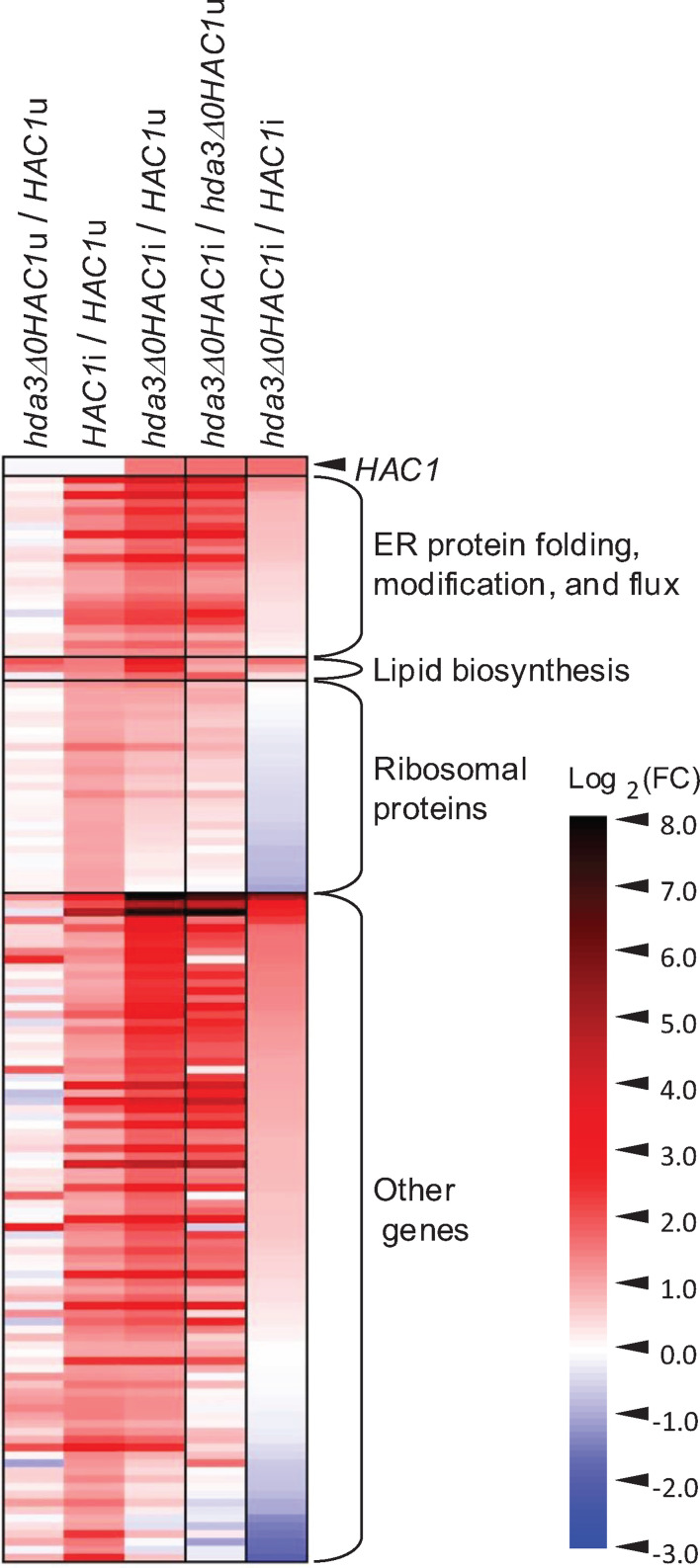
Expression profiles of *HAC1* and UPR-target genes. The mRNA-seq data (fold-TPM change) of *HAC1* and genes induced in *HAC1*i cells compared to *HAC1*u cells (*P* < 0.05, FC > 2.0) are presented as a heat map. The *HAC1*i mutation upregulated many ribosomal protein genes in the absence, but not in the presence of the *hda3*Δ mutation.

Figure 4 shows the expression profile of *HAC1* and the UPR target genes that were induced in *HAC1*i cells compared to *HAC1*u cells. *hda3Δ0HAC1*i cells expressed prominent UPR-target genes involved in ER protein folding, modification, and flux and in lipid biosynthesis (see Table S3 for the gene list) more strongly than *HAC1*i cells (reddish colored in the rightmost column). Nevertheless, the expression of ribosomal protein genes and some other genes was lower in *hda3Δ0HAC1*i cells than in *HAC1*i cells (bluish colored in the rightmost column). It should be noted that the expression of *HAC1* was higher in *hda3Δ0HAC1*i cells than in the other cell types.

### *hda3Δ0HAC1*i cells exhibit ER expansion and high-level biomolecule production

**Fig 5:**
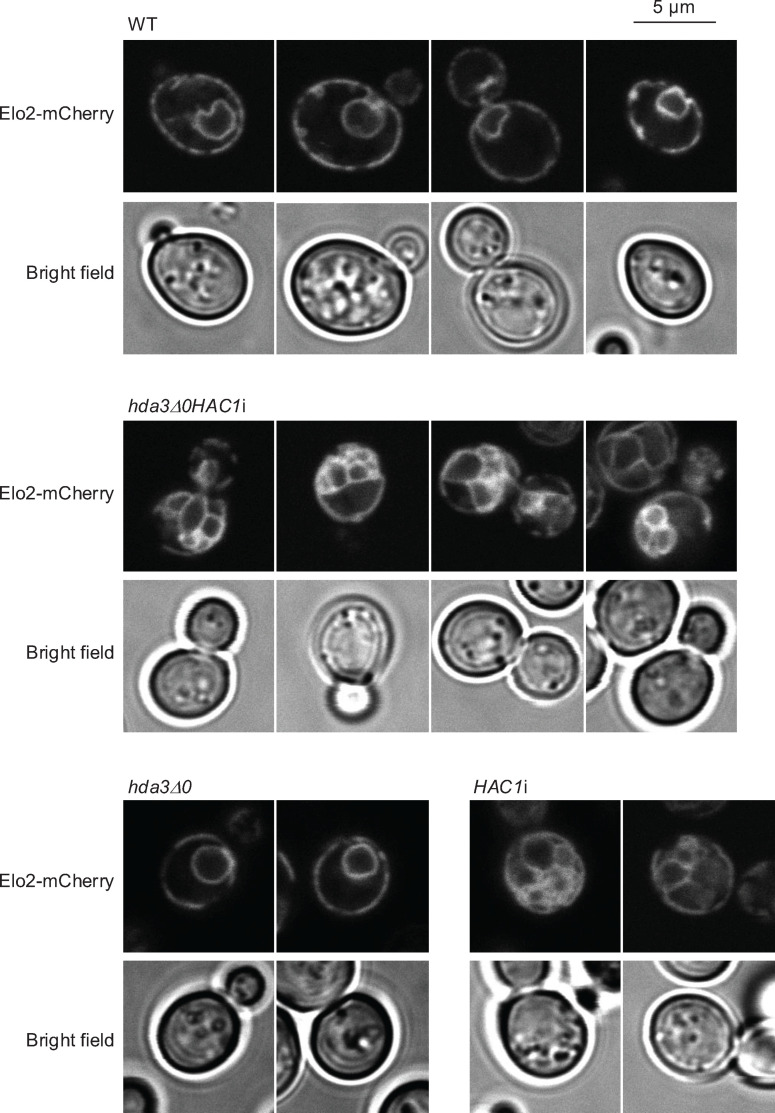
ER expansion in *hda3Δ0HAC1*i cells. Wild-type cells (WT; BY4742) and their derivative cells were transformed with the Elo2-mCherry expression plasmid pYT-TDH3p-ELO2-mCherry. The transformants were incubated at 30°C in SC liquid medium, and growing-phase cultures were observed under a confocal microscope. The ER apparently expanded in *hda3Δ0HAC1*i cells as well as in *HAC1*i cells.

We previously reported that ER is abnormally expanded in *S. cerevisiae* cells that highly express Hac1 (19, 20). Moreover, they bear more lipid droplets than wild-type cells (19, 20). Here, we investigated whether similar observations could be obtained from *hda3Δ0HAC1*i cells. In the experiments shown in Fig. 5, the ER was visualized by the expression of the mCherry-labeled ER-located protein Elo2 (Elo2-mCherry). The Elo2-mCherry image of wild-type cells showed a typical double-ring pattern representing the nuclear envelope and cortical ER. However, the ER appeared to be more expanded in *hda3Δ0HAC1*i cells. The ER expansion in *hda3Δ0HAC1*i cells is likely due to the *HAC1*i mutation, because it was also observed in *HAC1*i cells but not in *hda3Δ0* cells (Fig. 5).

**Fig 6:**
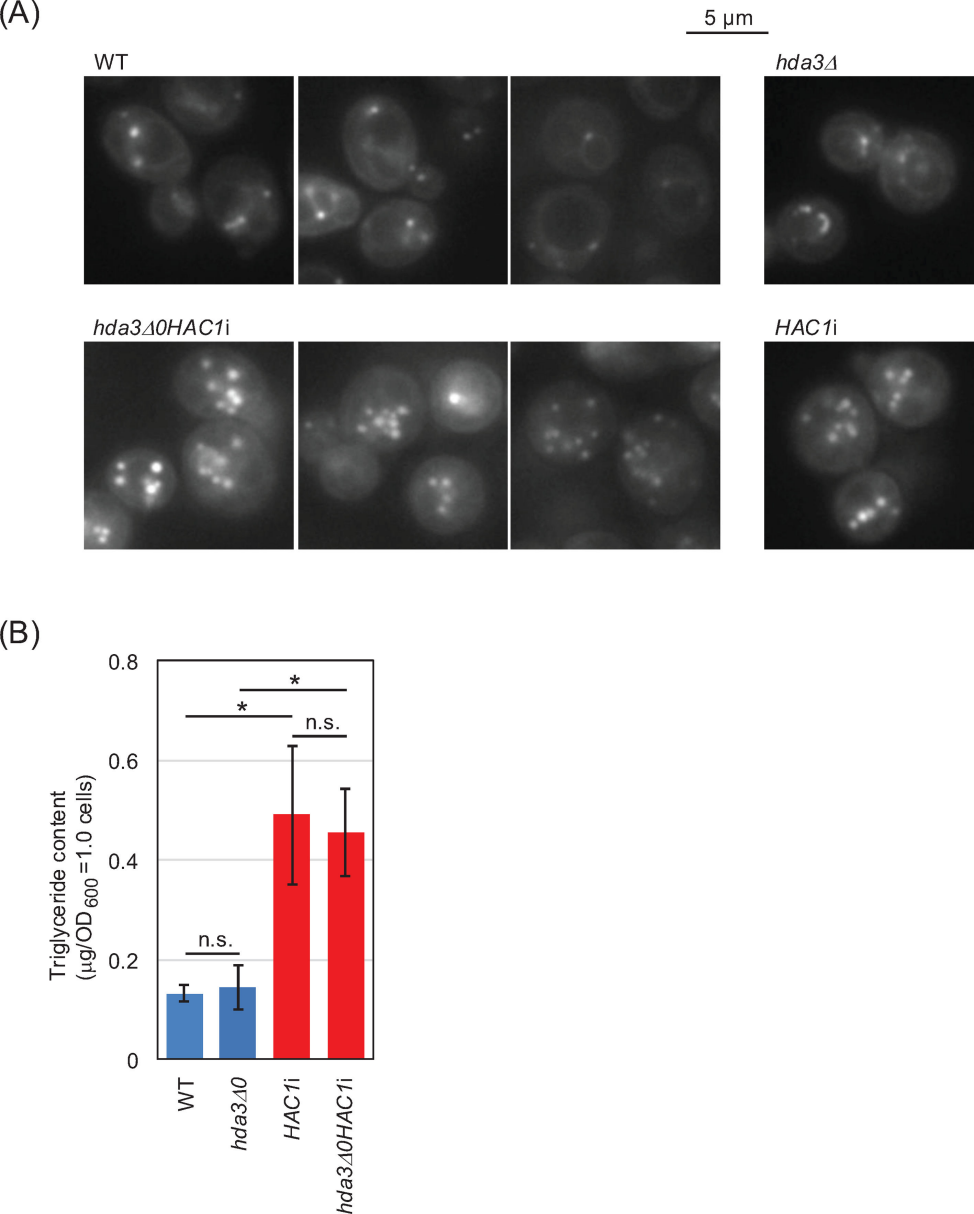
High accumulation of lipid droplets in *hda3Δ0HAC1*i cells. Wild-type cells (WT; BY4742) and their derivatives were incubated at 30°C in SC liquid medium until they reached the exponentially growing phase. (**A**) The cells were stained with BODIPY 493/503 and observed under a non-confocal fluorescence microscope. (**B**) Intracellular triglyceride levels were measured. Statistical significance was assessed by Tukey’s multiple comparison (n.s.: not significant; *P* > 0.05; *: *P* < 0.05). *hda3Δ0HAC1*i cells, as well as *HAC1*i cells, contained more neutral lipids than wild-type cells.

Furthermore, we stained the lipid droplets in which triglycerides were stored using the lipophilic fluorescent probe BODIPY 493/503. As shown in Fig. 6A, *hda3Δ0HAC1*i cells, as well as *HAC1*i cells, appeared to bear more lipid droplets than wild-type cells or *hda3*Δ cells. Figure 6B indicates that *hda3Δ0HAC1*i cells, as well as *HAC1*i cells, exhibited high triglyceride accumulation.

**Fig 7:**
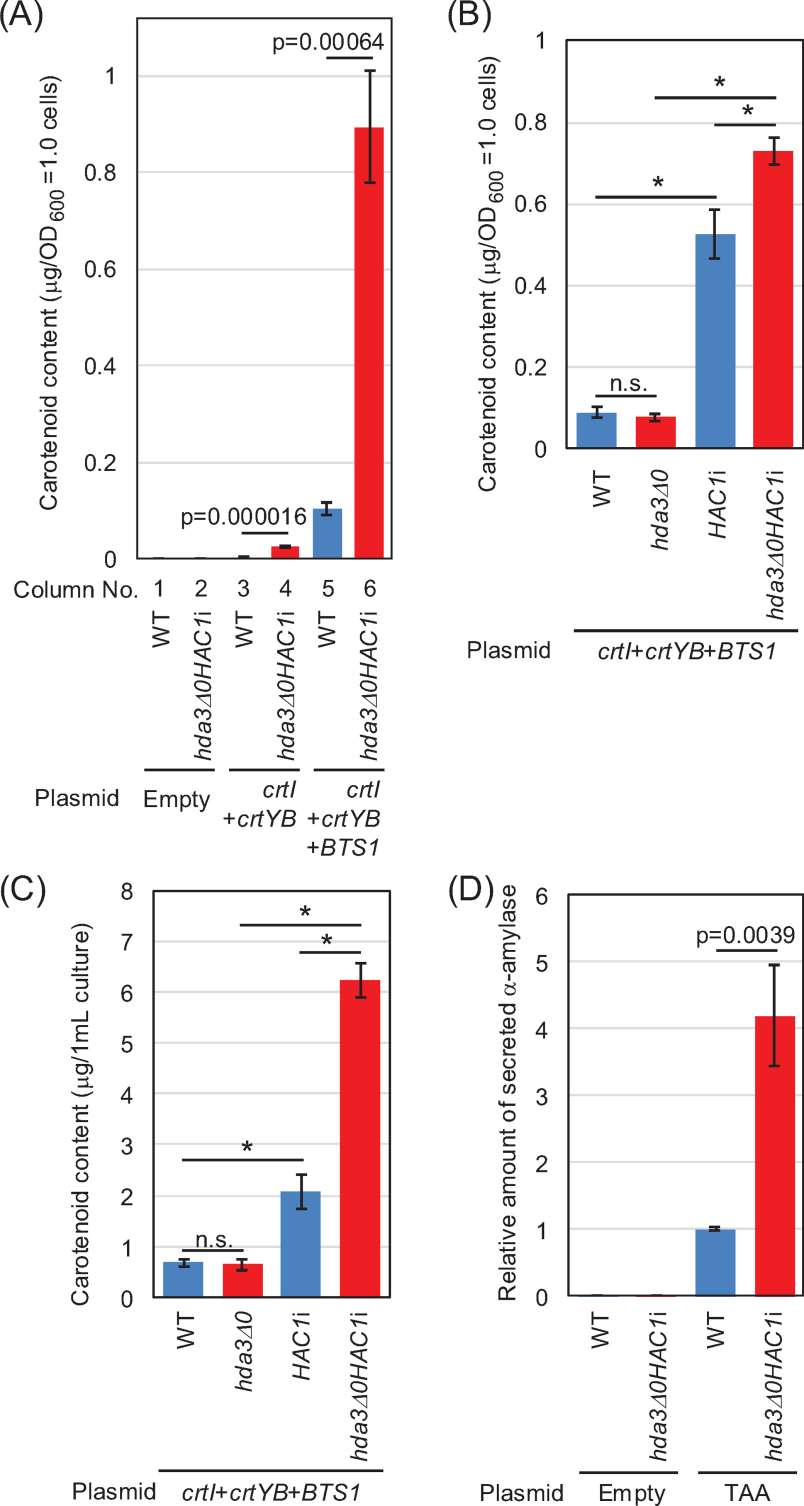
Increased production of heterologous biomolecules in *hda3Δ0HAC1*i cells. (A–C) Wild-type (WT; BY4742) cells and their derivatives were transformed with plasmids carrying the β-carotene genes, YEplac195-YB/I (*X. dendrorhous crtYB* and *crtI*) and YEplac195-YB/I/BTS1 (*X. dendrorhous crtYB*, *crtI*, and *S. cerevisiae BTS1*), or a control empty vector, pRS316. The transformants were incubated at 30°C in liquid SC medium for 24 h, and the intracellular β-carotene content was determined. (**D**) Wild-type (WT; BY4742) and *hda3Δ0HAC1*i cells were transformed with the TAA expression plasmid, pRS316TAA, or a control empty vector, pRS316. The transformants were incubated at 30°C in liquid yeast extract peptone dextrose (YPD) medium for 24 h, and the α-amylase activity of culture medium supernatants was determined. In A and D, statistical significance was assessed using Student’s *t*-test. In B and C, statistical significance was assessed using Tukey’s multiple comparison test (n.s.: not significant; *P* > 0.05; *: *P* < 0.05). *hda3Δ0HAC1*i cells produced heterologous biomolecules, β-carotene and TAA, more abundantly than wild-type cells when carrying plasmids for their production.

Next, an example of a heterologous and functional lipid molecule, β-carotene, was produced in *S. cerevisiae*. Considering future applications in industrial biomolecule production, the cells were grown to exit the exponential growth phase by incubation for 24 h before carotenoid measurement (see Fig. 2 for growth properties). *Xanthophyllomyces dendrorhous crtYB* and *CtrI* encode enzymes that constitute a metabolic pathway that converts geranylgeranyl diphosphate (GGPP) into carotenoids (24). As described by Verwaal et al. (28), the cells produced carotenoids, albeit slightly, when transformed with a plasmid to express *crtYB* and *CtrI* (Fig. 7A; columns 3 and 4). Notably, *hda3Δ0HAC1*i cells contained 3.6-fold more carotenoids than wild-type cells. Next, as done by Verwaal et al. (28), *S. cerevisiae BTS1*, which encodes GGPP synthase, was overexpressed together with *X. dendrorhous crtYB* and *CtrI*, resulting in higher production of carotenoids (Fig. 7A; compare columns 5 and 6 to columns 3 and 4). Also in this case, *hda3Δ0HAC1*i cells produced 8.2-fold more carotenoids than wild-type cells (Fig. 7A; columns 5 and 6).

In the experiment shown in Fig. 7B and C, wild-type, *hda3Δ0*, *HAC1*i, and *hda3Δ0HAC1*i cells were transformed with the plasmid to express *crtYB*, *CtrI*, and *BTS1*. As shown in Fig. 7B, carotenoid production in *HAC1*i cells was higher than that in wild-type or *hda3Δ0* cells but slightly lower than that in *hda3Δ0HAC1*i cells (1.4-fold difference between *HAC1*i cells and *hda3Δ0HAC1*i cells). Because *hda3Δ0HAC1*i cells grew better than *HAC1*i cells (see Fig. 2 for the growth properties), *HAC1*i and *hda3Δ0HAC1*i cells exhibited a more apparent difference (3.0-fold) in carotenoid production in Fig. 7C, in which the carotenoid yield was expressed on a per-culture-volume basis.

Finally, we monitored the production of heterologous secretory proteins using *Aspergillus oryzae* Taka-amylase A (TAA) as a model protein. Because SC medium is acidic and may cause denaturation of secreted proteins, we used a nearly neutral medium, yeast extract peptone dextrose (YPD). The cells were pre-cultured in SC medium to avoid plasmid loss, transferred to YPD, and incubated for 24 h to allow them to exit the fast-growing phase (Fig. S3). As shown in Fig. 7D, *hda3Δ0HAC1*i cells secreted 4.2-fold more TAA than wild-type cells.

We were unable to obtain *HAC1*i cells transformed with the TAA expression plasmid for unknown reasons. Such a difficulty in gene manipulation is also a disadvantage of *HAC1i* cells, which has been overcome in *hda3Δ0HAC1*i cells.

## DISCUSSION

Some yeast species, including *S. cerevisiae*, grow quickly and inexpensively, are easily subjected to genetic manipulation, and can be widely used for industrial bioproduction. The production of heterologous secretory proteins from genetically modified yeasts is well established in biotechnology (29). Lipid production by yeasts is also a noteworthy biotechnology. In the future, lipid molecules specifically produced by plants will also be industrially produced by yeasts. One advantage of lipid bioproduction by yeast is that, unlike plants, it does not require wide arable land. Because secretory proteins and many lipid molecules are produced in or on the ER, their production in *S. cerevisiae* can be improved by constitutive expression of Hac1 (11–13, 18–20). However, as described in the Introduction, the growth of *S. cerevisiae* cells that strongly express Hac1 is severely retarded (4, 19–21).

To overcome this drawback, we previously exposed Hac1-expressing cells to weak ER stress stimuli, leading to acceleration of their growth (19). However, because ER stress is harmful, it is difficult to grow cells under ER stress conditions for long periods. In another previous study, Hac1 was expressed from an inducible promoter (20). The expression of Hac1 was repressed in the seed culture and strongly induced at an appropriate time point. However, the culture procedure is complicated and may not be cost-effective.

In this study, we propose a new strategy for rapidly growing *S. cerevisiae* cells that artificially and constitutively express Hac1. We observed that the growth of Hac1-expressing cells was accelerated by HDA deficiency. Although many prominent UPR target genes were highly induced, *hda3Δ0HAC1*i cells grew faster than *HAC1*i cells. *hda3Δ0HAC1*i cells carried an expanded ER and exhibited high-yield production of triglycerides and heterologous biomolecules. Partly because of the difference in final cell density, the carotenoid yield from *hda3Δ0HAC1*i cells was considerably higher than that from *HAC1*i cells. Another advantage of *hda3Δ0HAC1*i cells is that they can be easily handled.

According to the transcriptome analysis performed in this study, many UPR target genes involved in ER function were more highly expressed by Hac1 in the presence than in the absence of the *hda3Δ0* mutation. This is another advantage of the *hda3Δ0* mutation. In general, histone acetylation relaxes the chromatin structure, leading to high accessibility of transcription factors to genomic DNA (27). Histone deacetylase B and HDA deacetylate histones in wide and distinct chromosomal regions (25, 26). Therefore, it is likely that HDA-deficient mutations, such as *hda3Δ0*, decrease the histone acetylation status and make Hac1 accessible to wider regions of genomic DNA. We also observed that Hac1 was more highly expressed in *hda3Δ0HAC1*i cells than in *HAC1*i cells. This may also be a cause of the high UPR induction in *hda3Δ0HAC1*i cells. Figure S1 shows that the *hda3Δ0* mutation elevated the acetylation status of histone H3, which is the major target of HDA, similarly in *HAC1*i cells and in *HAC1*u cells. Nevertheless, the *hda3Δ0* mutation affected a greater number of genes in *HAC1*i cells than in *HAC1*u cells (Fig. 3D and E). Therefore, we assume that the Hac1 overexpression and the histone acetylation synergistically affect the expression of many genes.

In contrast, we also noted that, although not large in number, there are genes induced (or repressed) in *HAC1*i cells, but not in *hda3Δ0HAC1*i cells. We speculate that this change in gene expression may explain why the growth retardation of *HAC1*i cells was rescued by the *hda3Δ0* mutation. *HAC1*i cells induced a substantial proportion of RPS and RPL family ribosomal protein genes that were suppressed by the *hda3Δ0* mutation. Therefore, it is possible that the growth retardation of *HAC1*i cells was caused by the aberrant expression of ribosomal protein genes. In agreement with this idea, Tye et al. (30) proposed that orphan and unassembled ribosomal proteins disturb cytosolic or nuclear proteostasis, leading to cellular damage in *S. cerevisiae*.

Ribosomes are assembled from the RPS and RPL family ribosomal proteins and rRNAs in the nucleus. A number of proteins, referred to as ribosome biogenesis factors, are known to assist in the biogenesis and assembly of ribosomes (31). It should also be noted that RPS and RPL genes belong to the group of most highly expressed genes in *S. cerevisiae* (Table S1). Therefore, we assume that orphan and unassembled RPS and RPL proteins may accumulate abundantly in the cytosol or nuclei when RPS and RPL genes are overexpressed without the induction of genes encoding ribosome biogenesis factors. This contrasts with the case of *P. pastoris*, in which many ribosome biogenesis genes, but not RPS and RPL genes, were reported to be induced by artificial overexpression of Hac1 (32). According to proteome analysis by Lin et al. (33), artificial overexpression of Hac1 decreased the cellular levels of some RPS and RPL proteins in *P. pastoris*. The outcomes of artificial overexpression of Hac1 likely differ somewhat between *S. cerevisiae* and *P. pastoris*.

What are the molecular mechanisms underlying ER expansion and high biomolecule production shown here? Based on the data from transcriptome analysis, we listed the genes that were induced or repressed in *S. cerevisiae* cells expressing Hac1 (Table S2). Scs3 is an ER-localized protein that has been reported to maintain phospholipid homeostasis and ER morphology (34, 35). Furthermore, Scs3 belongs to the fat storage-inducing transmembrane protein 2 (FIT2) family, which has enzymatic activity as an acyl-coenzyme A diphosphatase and is involved in lipid droplet formation (36). Thus, it is possible that, at least partly, ER expansion and high triglyceride accumulation in *hda3Δ0HAC1*i cells resulted from the induced expression of *SCS3*. In addition, other genes related to lipid biosynthesis and transport, such as *ARV1*, *ERG7*, *INA1*, and *RER2*, were also induced in *hda3Δ0HAC1*i cells.

As described in the Introduction, the ER membrane serves as a site for the biosynthesis of various lipid molecules. Lipid droplets bulge out of the ER membrane (37). Therefore, the high lipid production in *hda3Δ0HAC1*i cells may be a result of ER expansion. Thus, we anticipate that the strategy presented here can be applied to the production of various heterologous lipid molecules biosynthesized on the ER membrane. It is also possible that our strategy can be combined with other strategies that directly strengthen metabolic pathways for lipid biosynthesis. For instance, carotenoid productivity was elevated by the overexpression of *BTS1*, which is involved in the bioproduction of the carotenoid precursor GGPP at the ER membrane, not only in wild-type cells but also in *hda3Δ0HAC1*i cells.

Genes involved in protein folding, modification, and flux in the early secretory pathway, including those shown in Table S2, such as *ALG3*, *DPM1*, *ERJ5*, *ERO1*, *ERP1*, *ERP2*, *ERV29*, *EUG1*, *JEM1*, *KAR2*, *LHS1*, *MCD4*, *MPD1*, *OST2*, *PDI1*, *PMT1*, *PMT2*, *PMT3*, *SBH1*, *SCJ1*, *SEC12*, *SEC53*, *SEC59*, *SEC61*, *SEC62*, *SEC63*, *SEC66*, *SEC72*, and *SIL1*, are induced by Hac1. Therefore, it seems reasonable to assume that the cellular ability to produce secretory proteins is improved by artificial Hac1 expression. In the present study, we demonstrated an increase in heterologous protein secretion using TAA as a model product. Artificially Hac1-expressing *S. cerevisiae* cells have also been used to produce other secretory proteins (11–13). For example, Kim et al. (13) reported that the bioactivity of recombinant insulin secreted from yeast could be improved by artificial *HAC1* expression. As described in the Introduction section, the ability of Hac1 to elevate cellular bioproductivity correlates with its expression levels (11, 20). We anticipate that, by introducing an HDA-deficient mutation, it will be possible to easily handle and grow cells that strongly express Hac1, resulting in more efficient protein secretion.

In this study, we used *S. cerevisiae*, partly because it is a conventional model organism for which biological insights have been extensively accumulated. Nevertheless, other yeast species are or will also be used for the bioproduction of secretory proteins and lipid molecules. *K. phaffii* and *Yarrowia lipolytica*, as well as *S. cerevisiae*, are yeast species that belong to the *Saccharomycotina* subdivision. The production of heterologous proteins from *K. phaffii* is a widely used biotechnological technique (38). *Y. lipolytica* will also be used for this purpose. Moreover, *Y. lipolytica* and some other yeast species are oleaginous yeasts, which are promising candidates for industrial lipid production. Artificial Hac1 expression has also been reported to increase the bioproductivity of *K. phaffii* and *Y. lipolytica* (14–17). One implication of the current study is that mutations that affect chromosomal status can alter the characteristics of Hac1-expressing cells. We anticipate that the properties of yeast species other than *S. cerevisiae* that artificially express Hac1 will also be improved by introducing such mutations, including those in genes encoding histone modification enzymes.

## MATERIALS AND METHODS

### Yeast strains and plasmids

The *S. cerevisiae* strains used in this study were derived from the standard *S. cerevisiae* strains BY4742 and BY4741 (Table 1), which are isogenic to another standard strain, S288C (39). KanMX4-based gene knockout mutants of BY4742 or BY4741, namely Y11907, Y05347, Y03654, and Y05594 (Table 2), were obtained from EUROSCARF (http://www.euroscarf.de/).

**TABLE 1:** Yeast strains used in this study

Strain name	Genotype or description	Reference or source
BY4742	*MATα his3Δ1 leu2Δ0 lys2Δ0 ura3Δ0*	(39)
BY4741	*MATa his3Δ1 leu2Δ0 met15Δ0 ura3Δ0*	(39)
Y11907	*ire1Δ* (*ire1::kanMX4*) derivative of BY4742	EUROSCARF
Y05347	*hda1Δ* (*hda1::kanMX4*) derivative of BY4741	EUROSCARF
Y03654	*hda2Δ* (*hda2::kanMX4*) derivative of BY4741	EUROSCARF
Y05594	*hda3Δ* (*hda3::kanMX4*) derivative of BY4741	EUROSCARF
YMS001	*hda3Δ0* progeny of BY4742	This study
*HAC1*u strains (three independent clones)	*ire1Δ*/wild-type *HAC1* progenies of Y11907	(6, 19)
*HAC1*i strains (three independent clones)	*ire1Δ*/intronless *HAC1*i progenies of Y11907	(6, 19)
*hda3Δ0/HAC1*u strains (three independent clones)	*hda3Δ0*/*ire1Δ*/wild-type *HAC1* progenies of Y11907	This study
*hda3Δ0/HAC1*i strains (three independent clones)	*hda3Δ0*/*ire1Δ*/intronless *HAC1*i progenies of Y11907	This study

**TABLE 2:** Oligonucleotide primers used for construction of the *HDA3* 5′-UTR−loxP−*HIS3MX*−loxP−*HDA3* 3′-UTR DNA construct^*a*^

Oligonucleotide name	Sequence	PCR target
HDA3/−370F	CCCTATACCACTAAGGACGATAC	*HDA3* 5′-UTR (Forward)
P1	tggcggccgcgttcACTAAAGGGCAATTAGTTTGTGCTT (annealing to P2 [underlined region])	*HDA3* 5′-UTR (Reverse)
P2	attgccctttagtGAACGCGGCCGCCAGCTGAA (annealing to P1 [underlined region])	pUG27 (Forward)
P3	ttatttatttaatCTCCCCGCGCGTTGGCCGATTCAT (annealing to P4 [underlined region])	pUG27 (Reverse)
P4	caacgcgcggggagATTAAATAAATAATGCAG (annealing to P3 [underlined region])	*HDA3* 3′-UTR (Forward)
HDA3/+313R	CAACTAAATCAAATATTGGCG	*HDA3* 3′-UTR (Reverse)

^*a*^ The sequences hybridizing to the PCR targets are capitalized.

As in our previous studies (6, 19), a pop-in/pop-out scarless mutation (40) was performed to remove the *HAC1* intron sequence from the *S. cerevisiae* genome. For the pop-in step, Y11907 (*ire1D*) cells were transformed with the yeast integration plasmid pRS306-partial*HAC1*i, which carries the *URA3* selectable marker and the partial *HAC1*i gene. The resulting transformants carried duplicated *HAC1* genes (wild-type *HAC1* and partial *HAC1*i) at the *HAC1* locus. Pop-out progenies that carried either the original or the intronless *HAC1* gene were selected by *URA3* counter-selection using 5-fluoroorotic acid (Table 1; *HAC1*u strains or *HAC1*i strains). For *HAC1*i cells, we used freshly created clones because, as shown in our previous study (19), fast-growing and UPR-deficient mutants spontaneously appear and become dominant in continuous cultures.

To generate a marker-free *HDA3* deletion allele, *hda3Δ0*, we used the Cre-loxP system (41). The *HIS3MX* selectable marker placed between the two loxP sequences was carried on plasmid pUG27 (41). The YCp-type plasmid pSH68 was used to express the Cre protein under the *GAL1* promoter control (41). Using PCR (Pyrobest DNA polymerase; Takara Bio, Kusatsu, Japan) and Gibson DNA assembly (New England Biolabs, Ipswich, MA, USA), we generated a fusion DNA construct of *HDA3* 5′-UTR−loxP−*HIS3MX*−loxP−*HDA3* 3′-UTR. Table 2 lists the PCR primers used for this purpose. For the PCR DNA templates, we used pUG27 and yeast genomic DNA obtained from BY4742. To knock out the genomic *HDA3* gene, *S. cerevisiae* strains were transformed with the fusion DNA construct. The resulting transformants were further transformed with pSH68 and cultured in SC-galactose medium.

The YCp-type plasmid pYT-TDH3p-ELO2-mCherry (20) was used to express mCherry-labeled Elo2 from the constitutive and strong *TDH3* promoter. The YEp-type plasmid YEplac195-YB/I (28) was used to express *X. dendrorhous crtYB* and *crtI* under control of the *TDH3* promoter. The YEp-type plasmid YEplac195-YB/I/BTS1 (28) was used to express *X. dendrorhous crtYB*, *crtI*, and *S. cerevisiae BTS1* under control of the *TDH3* promoter. The YCp-type plasmid pRS316TAA was used to express TAA under control of the *TDH3* promoter (42). The YCp-type plasmid pRS316Hac1-238 carried the intronless *HAC1*i mutant version of the *HAC1* gene with its authentic 5′- and 3′-UTR (21). As an empty vector control, we used the YCp-type plasmid pRS316 (43), which, as well as the other plasmids described above, carried the *URA3* selectable marker.

### Yeast culturing and transformation

Unless otherwise noted, *S. cerevisiae* cells were grown at 30°C in SC medium containing 2% glucose, 0.66% Difco yeast nitrogen base (without amino acids; Thermo Fisher Scientific, Waltham, MA, USA), and a wide variety of amino-acid and vitamin supplements (44). To grow cells containing plasmids, SC medium was modified for auxotrophic selection. The medium was solidified with 2% BACTO agar (Thermo Fisher Scientific). Tunicamycin (Sigma-Aldrich) was added at a final concentration of 2 µg/mL. YPD medium contained 1% Difco yeast extract (Thermo Fisher Scientific), 2% Bacto peptone (Thermo Fisher Scientific), and 2% glucose.

For the spot assay, cell suspension (1.0 × 10^7^ cells/mL in phosphate-buffered saline [PBS]) was 10-fold serially diluted with PBS and spotted onto agar plates, which were incubated for 3 days.

The optical density (OD_600_) of the cultures was monitored using a spectrophotometer SmartSpec 3000 (Bio-Rad Laboratories, Hercules, California, USA). For liquid culture, cells were pre-cultured in SC medium, transferred to SC or YPD medium at an OD_600_ of 0.2–0.3, and further incubated under aerobic shaking conditions.

Transformation of *S. cerevisiae* cells was performed using the lithium acetate method (44).

### DNA analysis

Genomic DNA was extracted from *S. cerevisiae* cells using the Dr. GenTLE (from yeast) High Recovery kit (Takara Bio) and further purified using standard phenol/chloroform/isoamyl alcohol extraction and ethanol precipitation. The DNA samples were sent to GenWiz (South Plainfield, NJ, USA) for next-generation sequencing. The NEBNext Ultra DNA Library Prep Kit for Illumina (New England Biolabs) was used to construct libraries, which were then analyzed using an Illumina HiSeq instrument (Illumina, San Diego, CA, USA). Sequencing was performed using a 2 × 150 paired-end configuration.

The raw data were cleaned using Cutadapt (V1.9.1) and mapped to the reference genome using BWA (V0.7.17).

### RNA analysis

Total RNA was extracted from *S. cerevisiae* cells using the hot-phenol method and treated with DNase I to remove residual genomic DNA, as previously described (20). The RNA samples were sent to GeomeRead Co., Ltd. (Takamatsu, Japan) and subjected to poly T purification and high-throughput sequence analysis using a DNBSEQ-G400RS DNA sequencer (MGI Tech, Shenzhen, China; 2 × 150 bp paired-end reads, 1 Gb data/sample) (45). Raw FASTQ data were analyzed using CLC Genomics Workbench (Qiagen, Venlo, The Netherlands) for mapping and read counting. Genes that were not or marginally expressed (transcripts per million [TPM] = 0.00 in any clone) were eliminated from the data sets before further data processing.

### Microscopy

mCherry fluorescence was observed under a laser scanning microscope SP8 Falcon (Leica Microsystems, Wetzlar, Germany) using an HC PL APO 100×/1.40 oil STED white objective lens. The cells were illuminated using a 537 nm white light laser. The emitted photons were collected using internal Leica HyD hybrid detectors with a spectral window of 582–6,699 nm. The pinhole size was 1.00 Airy unit.

To visualize lipid droplets, *S. cerevisiae* cells were stained with the fluorescent dye BODIPY 493/503 (Sigma-Aldrich) and observed under a conventional fluorescence microscope, as previously described (20).

### Other techniques

After clarification by centrifugation (2,000 × *g*, 30 s), the culture media were analyzed for α-amylase activity using the α-amylase measurement kit (Kikkoman, Tokyo, Japan). The intracellular triglyceride and carotenoid levels were measured as previously described (19).

For western blot analysis to detect histone H3, cells were lysed under alkaline conditions, as described in Rossmann and Stillman (46). Cell lysates were subjected to standard SDS-polyacrylamide gel electrophoresis, followed by western blot analysis (47). To detect acetylated histone H3, rabbit polyclonal anti-histone H3ac (pan-acetyl) antibody (Active Motif, Carlsbad, CA, USA) was used as the primary antibody (1:1,000 dilution). To detect total histone H3, a rabbit polyclonal anti-histone H3 antibody (ab1791; Abcam, Cambridge, UK) was used as the primary antibody (1:1,000 dilution). Horseradish peroxidase-conjugated goat anti-rabbit antibody (Jackson ImmunoResearch Labs, West Grove, PA, USA) was used as the secondary antibody. After treating the blot membrane with enhanced chemiluminescence (ECL) reagents (Amersham ECL Western Blotting Detection Reagent; Cytiva, Marlborough, MA, USA), chemiluminescent signals were detected using the Chemiluminescence Imaging System FUSION (Vilber Bio Imaging, Marne-la-Vallée, France).

### Statistical analysis

We analyzed three independent clones of the same genotype and expressed the resulting values as averages and standard deviations from triplicate biological replicates. Unless otherwise noted, Student’s two-tailed unpaired *t*-tests were performed using Microsoft Excel to obtain *P*-values. For a multiple comparison, we performed Tukey’s test.

## Data Availability

Raw sequence data are available from the DNA Data Bank of Japan (https://ddbj.nig.ac.jp/search). The accession numbers for the genomic DNA sequence data are SAMD00803524 and SAMD00803525. The accession numbers for the mRNA-seq data are SAMD00803509, SAMD00803510, SAMD00803511, SAMD00803512, SAMD00803513, SAMD00803514, SAMD00803515, SAMD00803516, SAMD00803517, SAMD00803521, SAMD00803522, and SAMD00803523.

## SUPPLEMENTAL MATERIAL

Supplemental figures aem.00644-25-s0001.pdf Fig. S1 to S3.

Table S1 aem.00644-25-s0002.xlsx Total data of the mRNA-seq analysis.

Table S2 aem.00644-25-s0003.xlsx List of genes counted in the Venn diagrams shown in Fig. 3.

Table S3 aem.00644-25-s0004.xlsx Genes and their expression data presented in Fig. 4.

ASM does not own the copyrights to Supplemental Material that may be linked to, or accessed through, an article. The authors have granted ASM a non-exclusive, world-wide license to publish the Supplemental Material files. Please contact the corresponding author directly for reuse.
