# Supplementary material for: Potential of a constitutive-UPR and histone deacetylase A-deficient Saccharomyces cerevisiae strain for biomolecule production
Source: Appl Environ Microbiol. 2025 Aug 7;91(9):e00644-25. doi: 10.1128/aem.00644-25 (PMC12442391; doi:10.1128/aem.00644-25)
Supplement: Supplemental figures — Fig. S1 to S3. [file aem.00644-25-s0001.pdf]

Figure S1

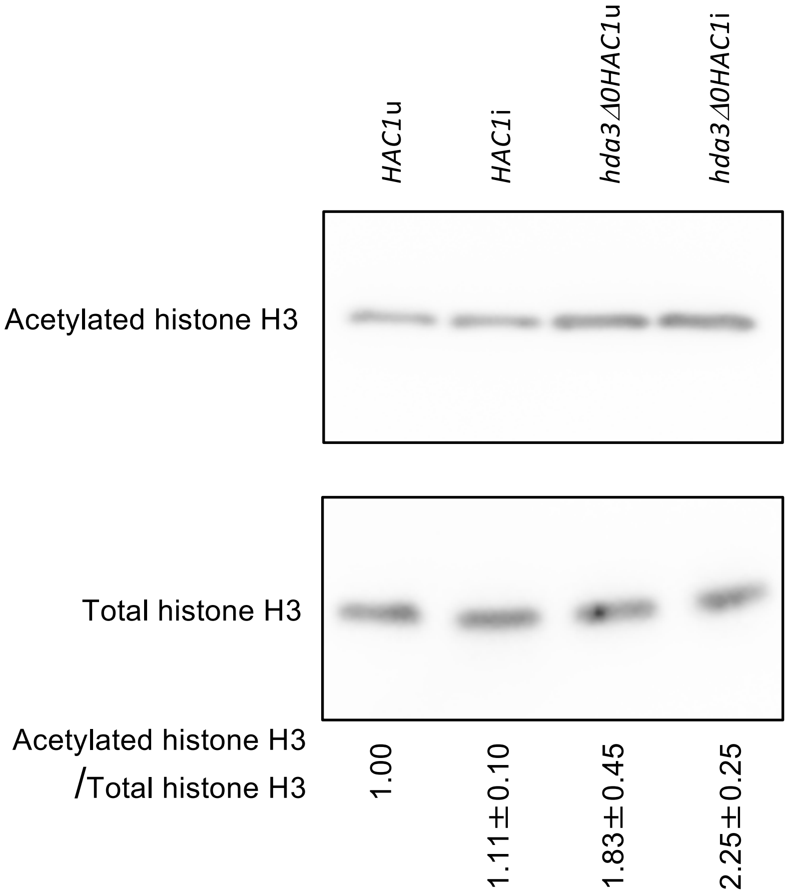

Figure S1 Effect of the *hda3Δ0* and *HAC1*i mutations on the histone H3 acetylation status

Cells were incubated at 30 ° C in SC liquid medium, and their lysates were subjected to western blotting to detect acetylated and total histone H3. The ratios of the ELC signal intensity of acetylated histone H3 to that of total histone H3 were normalized against that of *HAC1*u cells (set at 1.0) and are presented.

Figure S2

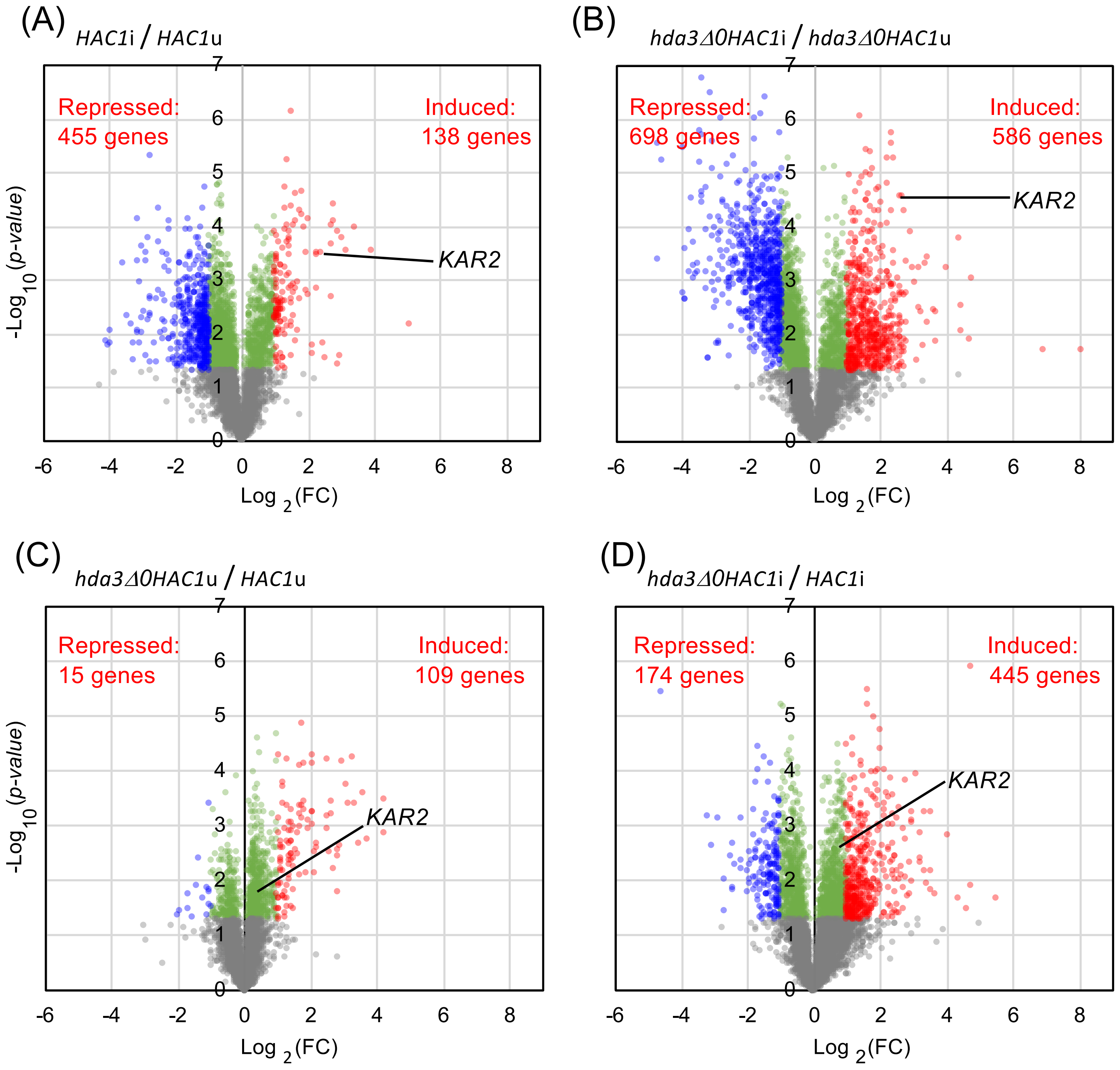

Figure S2 Volcano plots displaying DEGs between two cell types

Differences in TPM between the two cell types are expressed as volcano plots. The x-axis represents Log_2 of the FC, and the y-axis represents the negative decade logarithm of the p-value. DEGs ($p<0.05$, FC <–0.5 or >2.0) are indicated by red or blue spots. Other genes are indicated by green ($p<0.05$, 0.5< FC <2.0) or gray ($p>0.05$) spots.

Figure S3

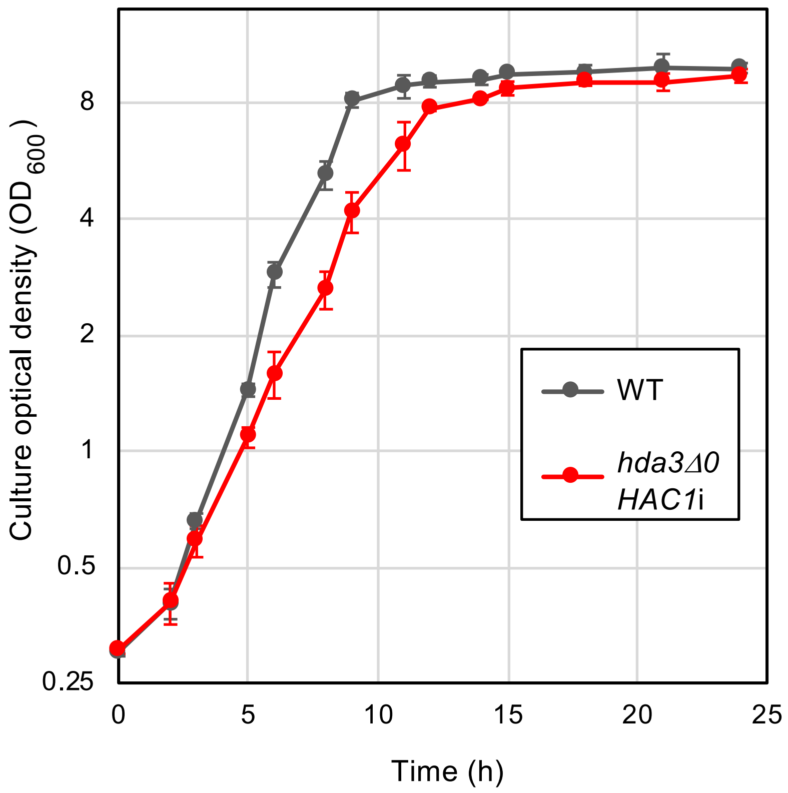

Figure S3 Growth profiles in YPD medium

Wild-type (WT; BY4742) and *hda3Δ0HAC1*i cells were grown at 30 ° C in YPD liquid medium, and the optical density of the cultures was monitored.
